# Baicalein inhibits PRRSV through direct binding, targeting EGFR, and enhancing immune response

**DOI:** 10.1186/s13567-024-01440-5

**Published:** 2025-01-20

**Authors:** Jing Wu, Qi Lu, Jing Hou, Yueqin Qiu, Min Tian, Li Wang, Kaiguo Gao, Xuefen Yang, Zongyong Jiang

**Affiliations:** 1 Institute of Animal Science, Guangdong Academy of Agricultural Sciences, Guangzhou, 510640 China; 2 State Key Laboratory of Swine and Poultry Breeding Industry, Guangzhou, 510640 China; 3 Key Laboratory of Animal Nutrition and Feed Science in South China, Ministry of Agriculture and Rural Affairs, Guangzhou, 510640 China; 4 Guangdong Key Laboratory of Animal Breeding and Nutrition, Guangzhou, 510640 China

**Keywords:** Piglets, PRRSV, baicalein, EGFR, MD simulation

## Abstract

Porcine reproductive and respiratory syndrome virus (PRRSV) presents significant economic challenges to the global pork industry due to its ability to mutate rapidly. The current commercial vaccines have limited effectiveness, and there are strict restrictions on the use of antiviral chemical drugs. Therefore, it is urgent to identify new strategies for preventing and controlling PRRSV infections. Baicalein, a flavonoid derived from *Scutellaria baicalensis*, has gained attention for its potential antiviral properties. However, there is little information about the effects and mechanisms of baicalein in relation to PRRSV. In this study, a network pharmacology analysis identified seven potential targets of baicalein against PRRSV, with the epidermal growth factor receptor (EGFR) emerging as the core target. The results of molecular docking and dynamics (MD) simulations confirmed that baicalein has a high binding affinity for EGFR, with a measured value of − 7.935 kcal/mol. Additionally, both in vitro (EC_50_ = 10.20 μg/mL) and in vivo (2.41 mg/kg) experiments were conducted to assess the effectiveness of baicalein against PRRSV. Notably, baicalein was found to inhibit various stages of the PRRSV replication cycle and could directly bind to PRRSV in vitro. Baicalein inhibited the entry of PRRSV by blocking EGFR phosphorylation and the downstream PI3K-AKT signaling pathway. This was confirmed by a decrease in the expression of p-EGFR/EGFR, p-AKT/AKT, PI3K, and SRC following treatment with baicalein. Additionally, baicalein significantly enhanced the immune response in piglets infected with PRRSV. In conclusion, this study suggests that baicalein may be a promising pharmaceutical candidate for preventing and controlling PRRS, offering new insights into the antiviral potential of Chinese herbal medicine.

**Supplementary Information:**

The online version contains supplementary material available at 10.1186/s13567-024-01440-5.

## Introduction

Porcine reproductive and respiratory syndrome (PRRS) is caused by the porcine reproductive and respiratory syndrome virus (PRRSV). It leads to respiratory distress in piglets and reproductive disorders in sows [1]. PRRS imposes a significant burden on the global swine industry, with annual losses in the USA reaching up to $664 million [2]. Therefore, it is essential to implement effective prevention and control measures for PRRS to improve breeding efficiency. PRRSV is a highly variable single-stranded RNA virus [3] that often undergoes recombination with other strains. This variability contributes to the slow development of effective vaccines against PRRSV [4]. Additionally, the excessive use of antiviral chemical drugs can pose risks to human health, food safety, and the environment [5]. Due to the limited effectiveness of current commercial vaccines and restrictions on the use of antiviral drugs, there is an urgent need to find a safe and effective strategy to prevent and control PRRSV in the swine industry.

Natural active ingredients found in plant extracts are significant because they typically have low toxicity, minimal side effects, and a reduced likelihood of drug resistance [6]. The primary flavonoid compounds of *Scutellaria baicalensis* include baicalin, baicalein, wogonoside, and wogonin [7]. These compounds perform a broad range of pharmacological activities, exhibiting anti-tumour, anti-inflammatory, antioxidant, and antiviral properties [8–11]. Notably, the antiviral effects of baicalein have been increasingly reported. Research has shown that baicalein inhibits the proliferation of the SARS-CoV-2 virus by binding to the non-catalytic site Cys145 on the M protein of the SARS virus. This interaction occurs through π-π stacking and hydrogen bonds [12]. Additionally, baicalein can bind to the catalytic core’s structural domain of human immunodeficiency virus (HIV) integrase, leading to conformational changes [13]. Moreover, baicalein has demonstrated efficacy against arboviruses such as Chikungunya Virus, West Nile Virus, Zika Virus, and Dengue Virus [10, 11, 14]. However, there is limited information regarding its effects on PRRSV.

This study utilised a combination of network pharmacology, molecular docking, and molecular dynamics simulations to identify EGFR as the core target of baicalein in its fight against PRRSV. Additionally, the anti-PRRSV effects of baicalein were examined through in vitro experiments with MARC-145 cells and in vivo animal experiments using weaning piglets. Additionally, the impact of baicalein on the EGFR-PI3K-AKT pathway in the lungs of piglets infected with PRRSV was confirmed. The results of this study suggest that baicalein is a promising pharmaceutical candidate for the prevention and control of PRRS and offers new insights into potential antiviral targets of plant extracts.

## Materials and methods

### Viruses, cell lines, and compounds

The PRRSV of genotype 2 (GD-ZJ, GenBank: MF772778.1, American type) and MARC-145 cells were generously provided by Professor Song’s lab [15]. MARC-145 is a cell line that is permissive to PRRSV, cultured in Dulbecco’s Minimum Essential Medium (DMEM; Gibco, UT, USA) with 10% fetal bovine serum (FBS; Biological Industries, Kibbutz Beit Haemek, Israel), along with 100 IU/mL penicillin, and 100 mg/mL streptomycin. The cells were maintained at 37 °C in an atmosphere containing 5% CO_2_. Baicalein was supplied by Zhucheng Haotian Pharmaceutical Co. LTD. (Shandong, China). The HPLC chromatogram results indicated that the purity of baicalein was 96.7% (Additional file 1).

### Animal experimental design, clinical observation, and sample collection

Weaned piglets (Duroc × Landrace × Yorkshire) with initial body weights of 7.4 ± 0.23 kg and an age of 21 days were randomly divided into three groups: ten piglets in the PBS group (PBS infection without baicalein treatment), ten piglets in the PRRSV group (PRRSV infection without baicalein treatment), and ten piglets in the PRRSV + Bai group (both PRRSV infection and baicalein treatment). There were equal numbers of males and females in each group of piglets. To prevent the spread of PRRSV, each group was kept separately.

Following the 2012 nutrient recommendations from the National Research Council (NRC), the basal diet served as the control diet (Additional file 2). The piglets in the PRRSV + Bai group received a basal diet supplemented with 2.4 mg/kg of baicalein. For a duration of two weeks, all three groups of piglets were fed four times a day: at 8:00, 11:00, 15:00, and 18:30. Then, intramuscular injections of 2 mL PRRSV (10^5^ TCID_50_/mL) were given to each piglet in the PRRSV and PRRSV + Bai groups.

Three weeks after PRRSV infection, necropsy and gross pathological examinations of the lungs were immediately performed upon each piglet following euthanasia with sodium pentobarbital (40 mg/kg body weight). During the experiment, the clinical symptoms of each piglet were monitored and evaluated using a detailed scoring system [16]. Daily, feed residues from each pen were collected and weighed, and the feed intake for each pen was recorded. The average daily feed intake (ADFI) was then calculated. To determine the average daily gain (ADG), all piglets were weighed at the beginning and end of the experiment after an overnight fast of 12 h. Two independent observers visually inspected each pig's faeces twice daily (8:00 and 14:00) along the feeding route. At the end of the experiment, serum and lung samples were collected with liquid nitrogen and stored at −80 ℃.

### Gene relative expression and absolute quantitation

Total RNA was isolated using TRIzol reagent (Invitrogen, Carlsbad, CA, USA). cDNA was synthesised from 1 µg of total RNA using the HiScript®IIQ RT Supermix Kit (Vazyme, China). The synthesised cDNA was then analysed by RT-qPCR following the manufacturer’s instructions, using the AceQ qPCR SYBR Green Master Mix (Vazyme). Primers listed in Additional file 3 were designed by Primer Premier 5.0. The qRT-PCR program consisted of an initial denaturation at 95 ℃ for 3 min, followed by 40 cycles of denaturation at 95 ℃ for 15 s, 58 ℃ for 15 s, and 72 ℃ for 35 s. The amplification products were analysed using the CFX Connect Real-Time System (Bio-Rad, CA, USA). The fold changes in the target genes were standardised against β-actin using the 2^ − ∆∆Ct^ method [17].

To determine the copy number of the virus, MARC-145 cells were infected with 100 TCID_50_ of PRRSV for 24 h. Total genomic DNA was extracted using the TIANamp Virus DNA/RNA Kit (TIANGEN, China). The copy number of the PRRSV-N was analysed using real-time PCR with an absolute quantitation method (the primers are listed in Additional file 3). Standard curves were established using plasmids with known copy numbers, allowing for the calculation of vector genome copy numbers based on this standard curve. The formula used for the copy number calculation was:

$${\text{Copy Number}}\, = \,\left( {{\text{amount}}\, \times \,{6}.0{2}\, \times \,{1}0^{{{23}}} } \right) \, / \, ({\text{length}}\, \times \,{1}0^{{9}} \, \times \,{66}0)$$

### Viral titre titration

At the end of each experiment, the supernatants from MARC-145 cells treated under different conditions were collected and subjected to a tenfold gradient dilution. We added 100 μL of the diluted supernatant to each well, with eight replicates per sample, and incubated them at 37 ℃ for 2 h. After incubation, the supernatant was removed and supplemented with fresh 2% FBS + DMEM. The cytopathic effect (CPE) of each well was documented at five days post-infection, and the 50% tissue culture infection dose (TCID_50_) was calculated using the Spearman-Kärber method.

### Western blotting analysis

Protein samples for western blotting analysis were separated using 12% SDS-PAGE gels and transferred onto polyvinylidene difluoride (PVDF) membranes with a pore size of 0.22 µm (Millipore, MA, USA) using a current of 160 mA for 90 min. The PVDF membranes were subsequently blocked with 5% (w/v) skimmed milk in TBST (10 mM Tris–HCl, 150 mM NaCl, 0.05% Tween-20; pH 7.4) for 1 h at 37 ℃. Following this, the membranes were incubated overnight at 4 ℃ in TBST containing 0.5% (w/v) skimmed milk with the following antibodies: PRRSV N protein antibody (1:500, Bioss, Beijing, China), EGFR/p-EGFR rabbit monoclonal antibody (1:2000, Cell Signaling Technology, MA, USA), PI3K antibody (1:1000, Proteintech, China), AKT/p-AKT antibody (1:1000, Proteintech, China), and SRC antibody (1:1000, Proteintech, China). The membranes were washed three times with TBST and then incubated for 1 h at 37 ℃ with HRP-conjugated goat anti-rabbit IgG (1:5000, Sigma, Aldrich, USA). After this incubation, the membranes were stained using ECL detection reagents (Thermo Fisher Scientific, MA, USA) following three additional washes with TBST. Finally, the blots were examined using a gel imager (Tanon, China).

### Antiviral activity assay

To assess the antiviral activity of baicalein, its cytotoxicity was first evaluated using a CCK-8 kit according to the manufacturer’s protocol (Beyotime, China). In brief, 96-well plates were seeded with 2 × 10^4^ MARC-145 cells in 100 µL. Gradient concentrations of baicalein or DMSO were then added at 37 ℃. After 48 h, 10 µL of CCK8 reagent was added to each well for 1 h. The whole procedure was performed whilst being shielded from light. The optical densities of the wells were measured at 450 nm using a microplate reader. Each experiment was conducted in triplicate. The cell growth rates were calculated using the formula: Cell growth rate (%) = (absorbance of treated cells/absorbance of the untreated control group) × 100. The 50% cytotoxic concentration (CC_50_) of baicalein was determined using GraphPad Prism 8.0.

The antiviral activity assay was conducted to evaluate the effectiveness of baicalein in inhibiting PRRSV in MARC-145 cells. MARC-145 cell monolayers in 96-, 24-, or 6-well plates were infected with 100 TCID_50_ of PRRSV in the essential medium for two hours at 37 ℃. Following the infection, the supernatants were removed, and fresh DMEM containing various concentrations of baicalein was added. After 48 h, both the cells and supernatants were collected for RT-qPCR, indirect immunofluorescence analysis (IFA), and western blotting. The concentration required for 50% of the maximal effect (EC_50_) of baicalein was constructed using GraphPad Prism 8.0, with ribavirin serving as the positive control.

### Time-of-addition assay

To determine the inhibitory kinetics, we conducted a time-of-addition experiment with minor modifications, as previously reported [18]. MARC-145 cells were grown to about 70% ~ 80% confluence in 24-well plates and then infected with 100 TCID_50_ PRRSV for 2 h at 37 °C. Baicalein was administered in conjunction with the PRRSV infection as a pre-treatment, co-treatment, or post-treatment approach. For pre-treatment, cells were incubated with baicalein for 6 h, 12 h, or 24 h at 37 °C. After this incubation, the cells underwent three washes with PBS before being infected with PRRSV for 2 h. Cells were treated concurrently with PRRSV and baicalein at 37 °C for 2 h. After removing the PRRSV-baicalein mixture, the cells were washed with PBS before fresh media was added. For post-treatment, the cells were treated with baicalein for 6 h, 12 h, or 24 h following the initial 2-h PRRSV infection at 37 °C. Subsequently, the cells were incubated in a fresh medium. Samples were collected 48 h after PRRSV infection, and the relative expression of the PRRSV ORF7 gene and the copy number of PRRSV were detected using qRT-PCR.

### Virus entry assay

1.5 × 10^5^ cells/well MARC-145 cells were seeded in 24-well plates and incubated at 37 ℃ for 6 ~ 12 h. Once the cells reached confluence, they were infected with 100 TCID_50_ PRRSV at 4 ℃ for 2 h. The unbound virus was removed using a citric acid buffer (composed of 40 mM KCl, 10 mM citric acid, 135 mM NaCl, pH 3.0) followed by washing the cells three times with PBS. After this, different concentrations of baicalein were added, and the cells were incubated at 37 ℃ for 2 h. Following another three washes with PBS, the cells were collected for virus load analysis using qRT-PCR.

### Direct interaction of baicalein with PRRSV

To determine whether baicalein directly interacts with PRRSV, a 100 TCID_50_ dose of PRRSV was mixed with varying concentrations of baicalein or ribavirin in an essential medium for 1 h at 37 °C. After this, PRRSV and baicalein were separated using ultrafiltration centrifugation. Specifically, an ultrafiltration device (0.5 mL, 30-kDa cutoff) was filled with the mixture of PRRSV and baicalein, followed by centrifugation at 7500 × *g* for 10 min at 4 °C. The resulting supernatant was washed repeatedly with medium to remove any leftover baicalein. The PRRSV particles that remained trapped in the ultrafiltration tube were then resuspended in essential fluid and allowed to infect MARC-145 cells, which were cultured in 6- or 24-well plates for 2 h. After washing the cells three times with PBS, they were cultured in fresh media for an additional 48 h at 37 °C before samples were collected to determine the PRRSV-infected cell count by IFA, and the PRRSV copy number by RT-qPCR.

### Identification of potential targets of baicalein in the fight against PRRSV

The 3D structure, PubChem-CID, and SMILES file of baicalein (CAS: 491-67-8) were obtained from the PubChem database [19]. The targets of baicalein were retrieved from several sources, including PharmMapper [20], Swiss Target Prediction [21], SEA Search Server [22], TCMSP [23], and STITCH online data library [24]. These targets were converted into gene symbols to create a network database using UniProt. Targets related to PRRSV were collected from two databases using the keyword “PRRSV”: the GeneCards database [25] and the Comparative Toxicogenomics database [26]. Potential targets for baicalein treatment of PRRSV were identified by intersecting the targets of baicalein with PRRSV-related targets. These results were then uploaded into Venny for mapping.

### GO and KEGG enrichment analysis and core target screening

To carry out GO and KEGG enrichment analyses, the potential targets of baicalein for the treatment of PRRSV were imported into the Database for Annotation, Visualisation and Integrated Discovery (DAVID) [27] with a significance threshold set at *P* < 0.05. The identified potential targets were then loaded into a protein–protein interaction (PPI) networks diagram using the Search Tool for the Retrieval of Interacting Genes/Proteins (STRING) database [28]. *Sus scrofa* was selected as the organism, and a minimum interaction score of 0.9 was established as the requirement. For core target screening and visualisation, the TSV-format file was retrieved from the STRING database and imported into Cytoscape 3.7.2 [29].

### Molecular docking

The molecular affinity of baicalein (Compound CID: 5281605) for the protein target EGFR (Uniprot ID: A0A5G2QBP1) was investigated using molecular docking techniques. Protein crystal structures were obtained from the PDB database, while the 3D structure of baicalein was downloaded from the PubChem database [19].

Molecular docking was carried out using the AutoDock 3.10.0 program, and the PyMoL 2.5 software was used to preprocess all receptor proteins by removing small molecules, salt ions, and water molecules before docking [30]. All processed baicalein, EGFR, and Gp5/M were converted into the PDBQT format required for docking with the help of OpenBabel 2.3.2 [31].

The binding conformation obtained from molecular dynamics simulations was determined by selecting the structure that exhibited the highest molecular docking score. To assess this, the original crystal ligand was redocked with the protein, allowing for an examination of the binding site poses, as well as the chemical bond lengths and chemical bond angles of the crystal ligand, which were all compared to the protein target. This analysis used the original crystal ligand as a positive reference. Ultimately, the accuracy of the molecular docking results can be evaluated based on the consistency of the binding mode.

### Molecular dynamics simulation (MD simulation)

The Groningen Machine for Chemicals Simulations (GROMACS) 5.0 software [32] and the CHARMM36 force field [33] were employed for molecular dynamics (MD) simulation based on docking data, under periodic boundary conditions for the molecules. Ligand topology files were generated using the CHARMM General Force Field [34], and ions were added neutralise the system’s charge. To minimise energy and eliminate any close interactions, the steepest-gradient approach was utilised. The Van der Waals and electrostatic interactions, along with energy calculations, were carried out using the particle mesh Ewald (PME) methods. Initially, the systems were allowed to acclimate for 50 000 steps in the NVT ensemble, followed by an additional 50 000 steps in the NPT ensemble. Finally, molecular dynamics simulations were conducted for either 50 or 100 ns at 300 K, using a time step of 2.0 fs. Coordinates were stored for analysis every picosecond.

### Statistical analysis

Data were analysed using GraphPad Prism 8.0 and presented as mean ± SEM for animal experiments and mean ± SD for in vitro experiments using IBM SPSS Statistics V26.0.0 (IBM Corp., Armonk, NY, USA). The Shapiro–Wilk test was conducted to assess whether the distribution was normal for all variables. A comparison of mean values between the groups was performed using one-way ANOVA followed by the Tukey post hoc test, with a significance level set at *P* < 0.05.

## Results

### Analysis of network pharmacology and targets of baicalein against PRRSV

**Figure 1:**
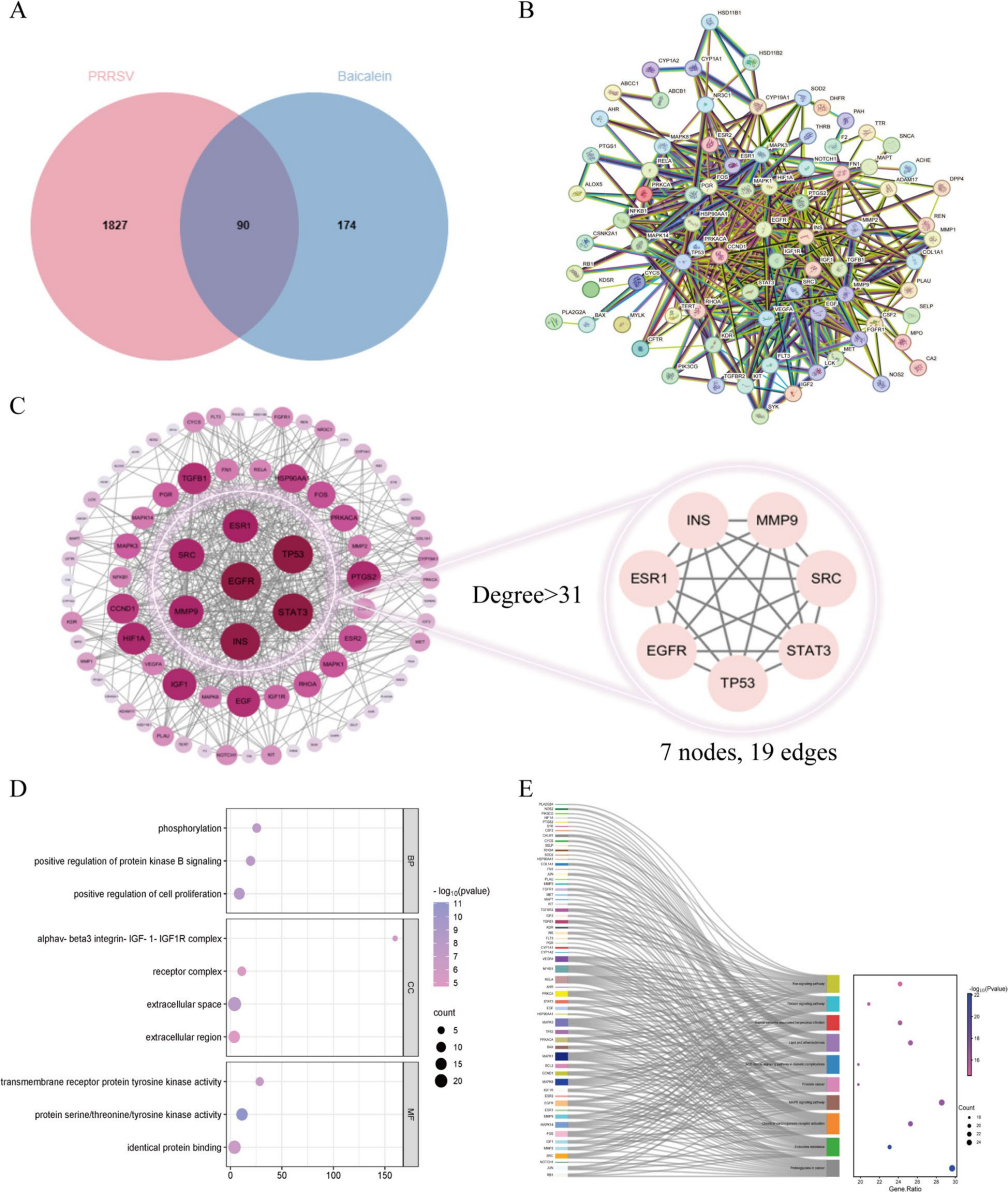
**Network pharmacology and targets analysis of baicalein against PRRSV**. **A** Interaction analysis of target genes between baicalein and PRRSV-related. **B** PPI network analysis. 90 potential targets were input into a String database, and a PPI network was constructed (*P*-value < 1.0^e−16^). **C** Topological analysis by Cytoscape 3.10.0. The seven core targets were screened and highlighted in the right box. The core targets screening criteria were set as degree > 31. **D **GO enrichment analysis of the 90 potential targets. **E** Top 10 of KEGG signalling pathway analysis of the 90 potential targets. The size of the dot represents the counts of proteins in this pathway; the colour of the dot corresponds to different *P*-value ranges.

264 potential targets of baicalein were identified from various online databases, including the PharmMapper, Swiss Target Prediction, SEA Search Server, TCMSP, and the STITCH online data library. Additionally, 1917 PRRSV-related targets were identified using the Comparative Toxicogenomics Database (CTD) and GeneCards database. By intersecting the targets of baicalein with the PRRSV-related targets, we found 90 overlapping potential targets, highlighting the effectiveness of baicalein against PRRSV (Figure 1A).

The 90 potential targets were input into the String database with the “Organisms” parameter set to “*Sus scrofa*”, resulting in the generation of a protein interaction network. In the String online database, a Protein–Protein Interaction (PPI) network was constructed (Figure 1B), which consisted of 86 nodes and 522 edges. The average node degree was calculated to be 12.1 (*P*-value < 1.0^e−16^). Subsequently, a topological analysis of the network was conducted using Cytoscape 3.10.0 software, leading to the establishment of criteria for identifying core targets. A threshold of degree > 31 was set, resulting in the identification of seven core targets, interconnected by 19 edges: EGFR, TP53, STAT3, SRC, MMP9, INS, and ESR1 (Figure 1C).

The set of 90 potential targets was analysed using the DAVID database to obtain Gene Ontology (GO) enrichment results. The subsequent examination of GO terms (Figure 1D) highlighted 333 functional clusters primarily associated with “phosphorylation”, “receptor complex”, and “transmembrane receptor protein tyrosine kinase activity”. These clusters were categorised into three main areas: Biological Process (BP), Cellular Component (CC), and Molecular Function (MF). Additionally, a KEGG pathway analysis generated a total of 164 pathways. After sorting these pathways based on *P*-values, the top 10 signalling pathways were selected for visualisation (Figure 1E). Importantly, the targets related to the anti-PRRSV activity of baicalein showed significant enrichment in pathways such as the “MAPK signalling pathway”, “Lipid and atherosclerosis”, and the “Ras signalling pathway”.

### Molecular docking and MD simulation of baicalein with EGFR

**Figure 2:**
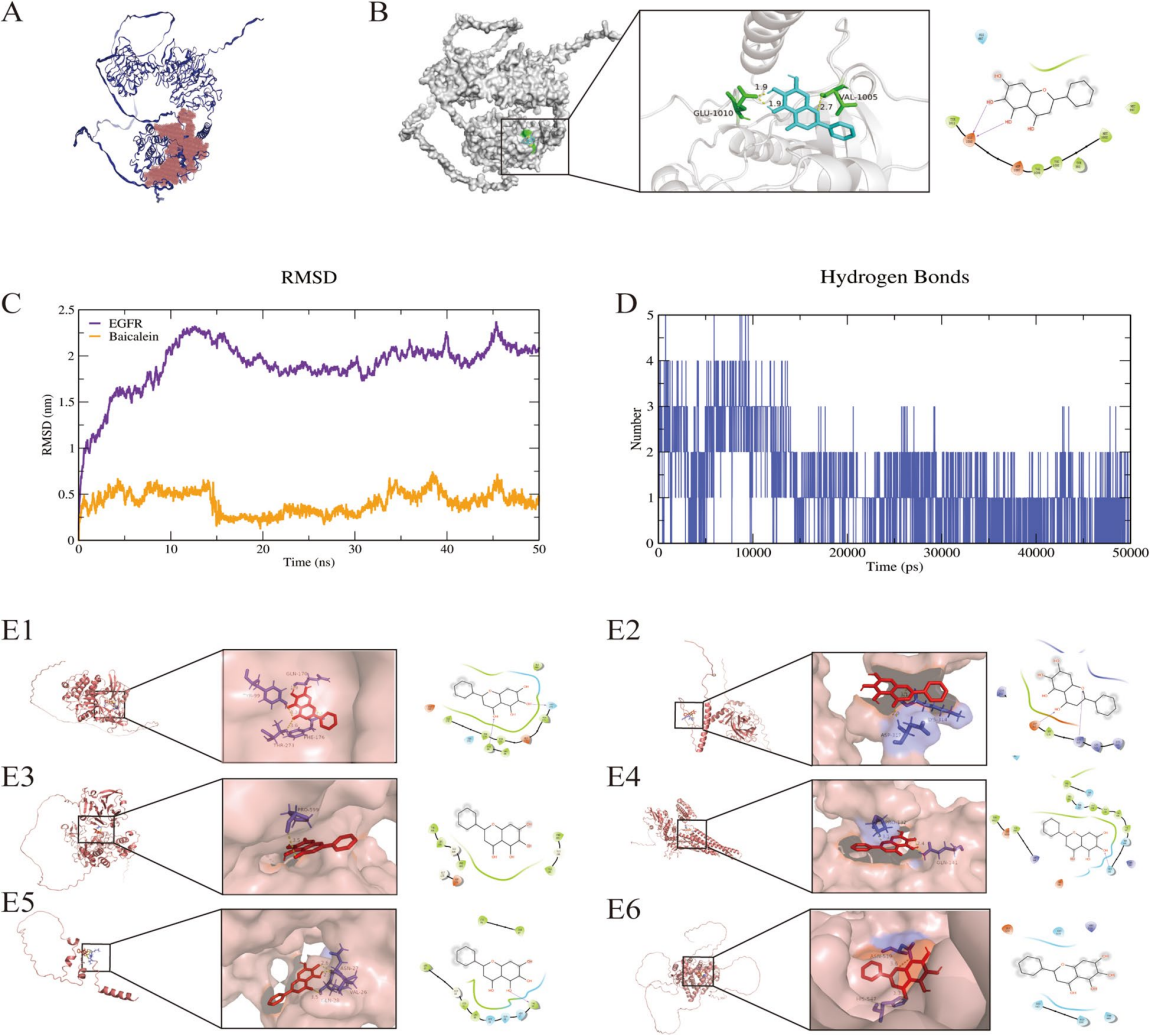
**Molecular docking and MD stimulation of baicalein with EGFR**. **A** Active pocket of EGFR prediction. The volume of the EGFR binding pocket was calculated with CHECOM algorithm by Yinfo technology. Active pocket of EGFR was shown in red with 25,465 Å^3^.** B** Molecular docking of EGFR (Uniprot ID: A0A5G2QBP1) with baicalein (Compound CID: 5281605) in a 3D model and 2D ligand interaction diagram. **C** Root Mean Square Deviation (RMSD) of baicalein and EGFR during molecular dynamics simulation. **D** Hydrogen bond numbers between baicalein and EGFR at different times. **E** Molecular docking of baicalein with another six core protein targets. E1-E6: SRC, TP53, MMP9, STAT3, INS, and ESR1.

Using AutoDockTools 1.5.6 software, molecular docking was performed to investigate the binding interactions between baicalein and seven potential protein targets at a molecular level. Baicalein was docked individually into the active sites of these core target proteins, and the resultant theoretical binding modes were visualised using PyMOL and Schrodinger software (Table 1 and Figure 2E). The results indicated that among the seven binding sites analysed, the epidermal growth factor receptor (EGFR) demonstrated the highest binding affinity for baicalein, as reflected by the lowest binding energy of − 7.935 kcal/mol. The key binding residues were identified as GLU-1010 and VAL-1005 (Figures 2A and B). Additionally, MD simulations conducted on the EGFR-baicalein complex further supported the strong binding capability of baicalein to EGFR (Figures 2C and D).

**Table 1:** **Binding of baicalein with potential targets**

Target	Common name	Uniprot ID	Affinity (kcal/mol)	Interact amino acid residues
Epidermal growth factor receptor	EGFR	A0A5G2QBP1	−7.935	GLU-1010, VAL-1005
Tyrosine-protein kinase	SRC	K7GPR7	−3.64	GLN-170, TYR-99
Cellular tumour antigen p53	TP53	Q9TUB2	−5.35	LYS-314, ASP-317
Matrix metalloproteinase-9	MMP9	A0A287AZD5	−2.61	PRO-599
Signal transducer and activator of transcription 3	STAT3	Q19S50	−2.68	PRO-132, GLN-141
Insulin	INS	P01315	−3.60	ASN-27, VAL-26, GLN-28
Oestrogen receptor	ESR1	Q29040	−3.15	ASN-519, HIS-547

### Antiviral effect of baicalein on PRRSV infection in vitro

**Figure 3:**
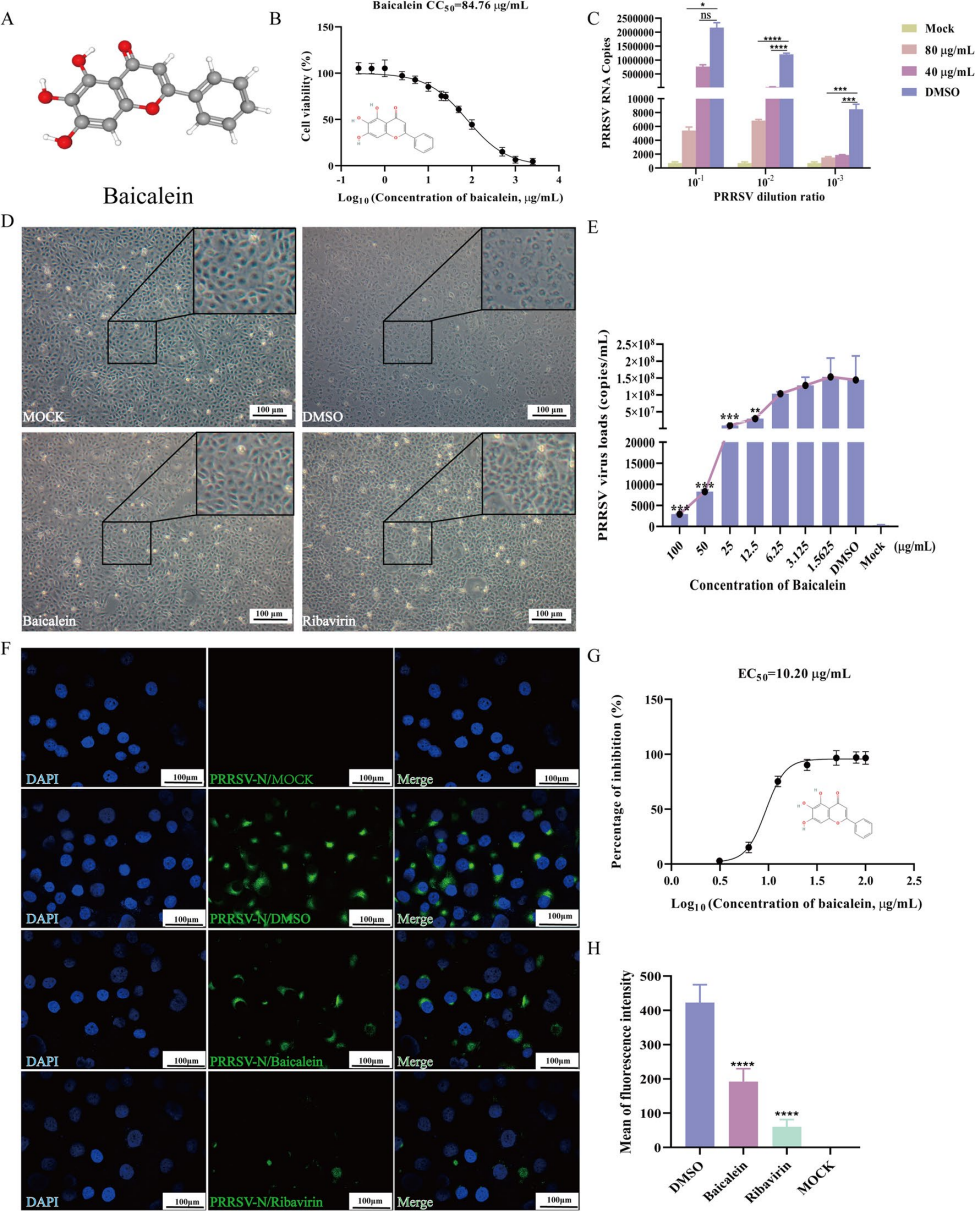
**Antiviral effect of baicalein on PRRSV infection in vitro**. **A** 3D chemical structure of baicalein. **B** 50% cytotoxic concentration (CC_50_) analysis. **C** Copies number of 10^–1^, 10^–2^, 10^– 3^ fold dilution PRRSV. **D** CPEs of MARC-145 cells. MARC-145 cells were treated with DMSO, baicalein (80 μg/mL), and ribavirin (120 μM), respectively, and infected with PRRSV (100 TCID_50_). PBS was used as a mock infection in the negative group. Scale bar = 100 μm. **E** The antiviral effect of baicalein on PRRSV was examined through the calculation of viral copies at various concentration gradients. **F** Immunofluorescence assay of protein N. Scale bar = 100 μm.** G **EC_50_ of baicalein against PRRSV was constructed by GraphPad prism 8.0. The data are expressed as mean ± SD. Each experiment was performed with three biological replicates and three technical replicates.** H** Mean fluorescence intensity (Mean ± SEM, *n* = 3). *P < 0.05; **P < 0.01, ***P < 0.005, ****P < 0.0001 compared with the DMSO treatment group.

To ensure the safety of baicalein for cellular use, we assessed its cytotoxicity in MARC-145 cells using a CCK-8 assay with DMSO at concentrations < 1%. The results indicated that baicalein exhibited dose-dependent cytotoxicity in these cells, with a 50% cytotoxic concentration (CC_50_) of 84.76 μg/mL (Figure 3B).

To further explore the antiviral effects of baicalein on PRRSV infection, we first examined the morphological changes in MARC-145 cells infected with PRRSV (100 TCID_50_) after treatment with baicalein at a concentration of 80 μg/mL, observing the changes under a microscope. Ribavirin (120 μM), known as an anti-PRRSV mutagen [35], served as a positive control in our experiments. PRRSV-infected MARC-145 cells displayed typical cytopathic effects (CPE) including cell shrinkage, rounding, and fusion with cytoplasmic vacuolisation. These effects were reduced by treatment with baicalein and ribavirin (Figure 3D).

We also investigated the antiviral effects of baicalein on PRRSV at dilutions of 10^–1^, 10^–2^, 10^–3^ fold. Although low-dose baicalein did not inhibit high concentrations of PRRSV infection, it exhibited significant dose-dependent antiviral activity between concentrations of 12.5 and 100 μg/mL (*P* < 0.05) (Figures 3C and E). Results from the Immunofluorescence assay demonstrated that baicalein could suppress the expression of the PRRSV-N protein (Figures 3F and H). Additionally, by calculating the viral copies, we determined the fifty percent effective concentration (EC_50_) value for baicalein against PRRSV infections to be 10.20 μg/mL (Figure 3G). These results suggest that baicalein has the potential to inhibit PRRSV, with its antiviral effect being dose and toxicity dependent.

### Baicalein inhibits PRRSV proliferation in pre-, co-, and post-treatment modes

**Figure 4:**
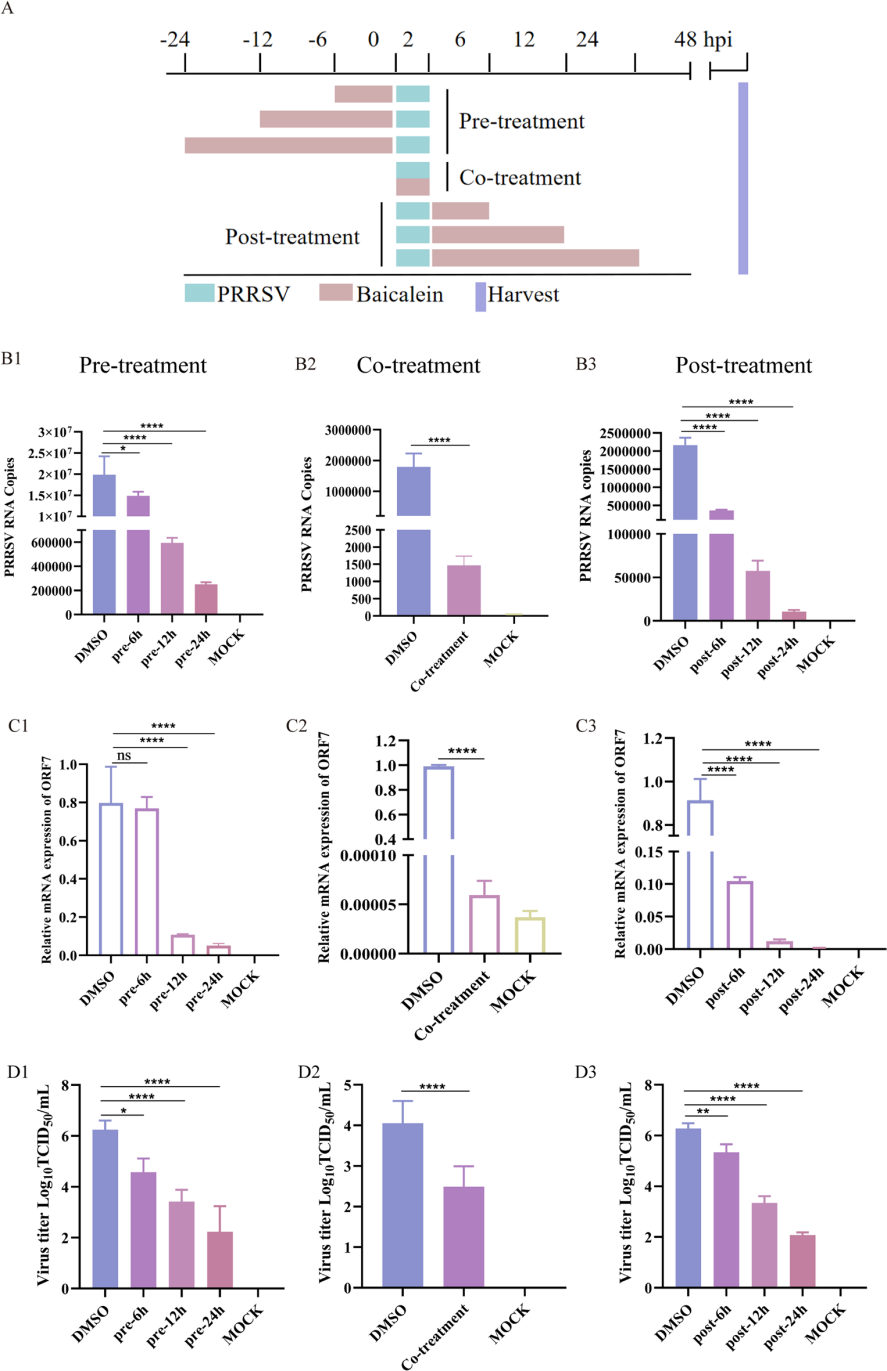
**Baicalein inhibited PRRSV proliferation in pre-, co-, and post-treatment modes in MARC-145 cells**. **A** Schematic diagram of time-of-addition assay. Briefly, MARC-145 cells were infected with PRRSV (100 TCID_50_), and the cells were treated with 80 μg/mL baicalein for different times before (pre-treatment), during (co-treatment) or post-infection (post-treatment), as shown in the schedules. At 48 hpi, the cells and supernatants were harvested for analysis.** B** PRRSV copy number calculation using absolute quantification.** C** PRRSV-ORF7 relative expression analysis using qRT-PCR. **D** Virus titres of PRRSV. TCID_50_ of PRRSV was calculated by the Spearman-Kärber method. Each experiment was performed with three biological replicates and three technical replicates. The data are expressed as mean ± SD. *, *P* < 0.05; **, *P* < 0.01, ***,* P* < 0.005, ****, *P* < 0.0001 compared with the DMSO treatment group.

To investigate the specific phase of the PRRSV replication cycle during which baicalein exerts its antiviral effects, a time-of-addition assay was conducted. In this assay, MARC-145 cells were exposed to PRRSV for a 2 h duration while baicalein was administered before (pre-treatment), at the same time (co-treatment), and after (post-treatment) the PRRSV infection, each for varying incubation times (Figure 4A).

Notably, pre-treatment with baicalein for periods ranging from 6 to 24 h resulted in a significant reduction in the copy number of PRRSV, the relative expression of PRRSV’s ORF7 mRNA and the virus titer in a time-dependent manner (Figures 4B1, C1, D1). These findings highlight the substantial inhibitory effect of baicalein pre-treatment during this time period on PRRSV infection in MARC-145 cells.

Intriguingly, administering baicalein during PRRSV infection (co-treatment) and after PRRSV infection also led to a significant and time-dependent decrease in the PRRSV copy number (Figures 4B2, B3), the relative expression of PRRSV’s ORF7 mRNA (Figures 4C2, C3) and the virus titer (Figures 4D2, D3). Overall, these results indicate that baicalein effectively suppresses PRRSV replication in MARC-145 cells both before, during, and following viral infection.

### Baicalein directly interacts with PRRSV

The significant inhibition of PRRSV infection by baicalein, as demonstrated, was observed even when the virus was not present (pre-treatment mode). This finding has prompted speculation about the potential inhibitory mechanisms of baicalein, possibly involving its interaction with cellular components. However, it has not yet been definitively established whether baicalein directly interacts with PRRSV.

**Figure 5:**
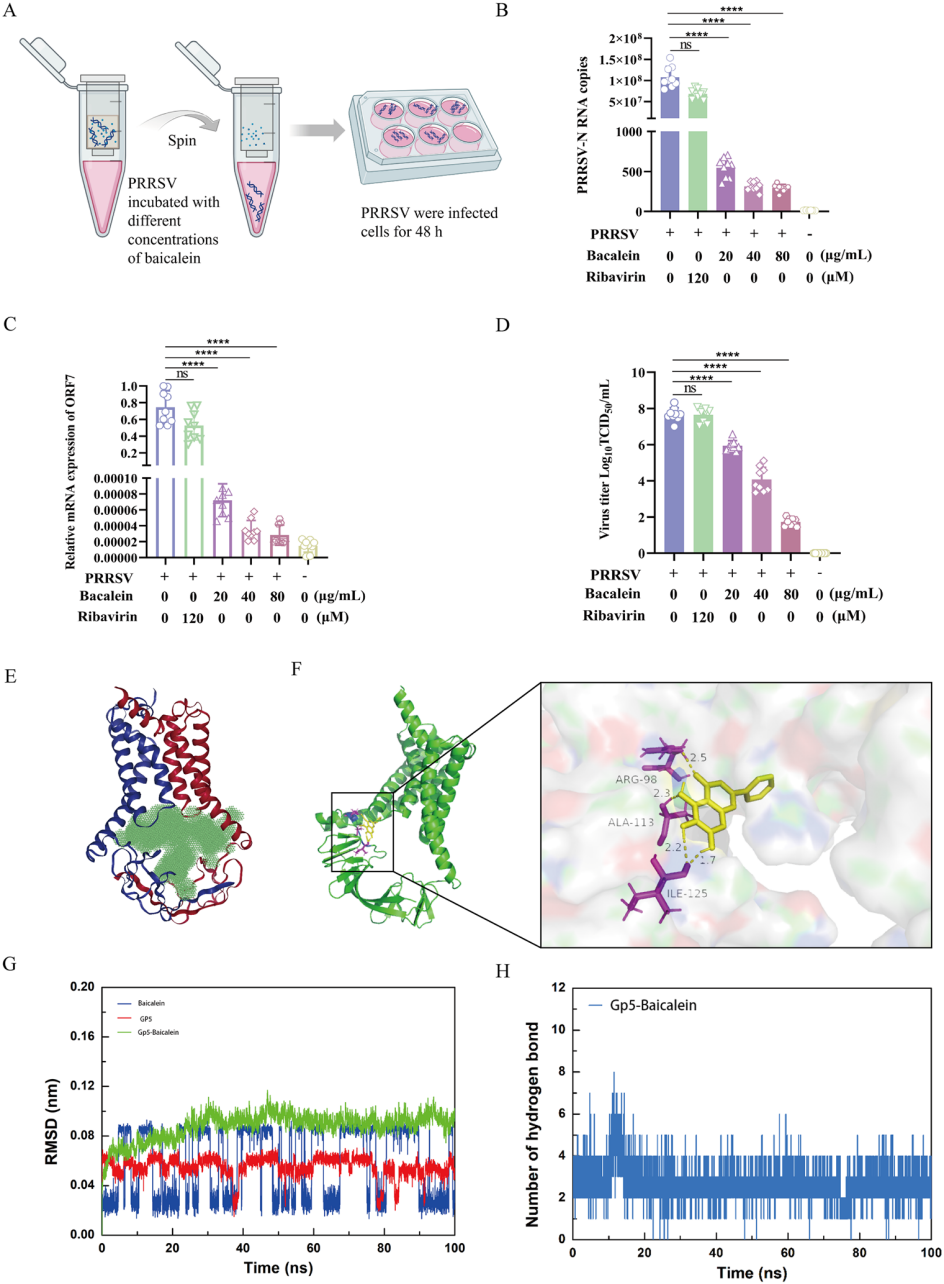
**Baicalein directly interacted with PRRSV**. **A** Schematic diagram of experimental design. Briefly, PRRSV (100 TCID_50_) was subjected to incubation with baicalein at different concentrations (20, 40, and 80 μg/mL) in essential medium for a duration of 1 h at 37 °C. Following this incubation, PRRSV was isolated from baicalein through the process of ultrafiltration. The retrieved PRRSV was then challenged to MARC-145 cells. Subsequently, at 48 hpi, the cells were collected for analysis. **B** PRRSV copy number calculation using absolute quantification. **C** PRRSV-ORF7 relative expression analysis using qRT-PCR. **D** Virus titres of PRRSV. TCID_50_ of PRRSV were calculated by the Spearman-Kärber method. Each experiment was performed with three biological replicates and three technical replicates. The data are expressed as mean ± SD. *P < 0.05; **P < 0.01, ***P < 0.005, ****P < 0.0001 compared with the only PRRSV treatment group. **E** Active pocket of Gp5/M prediction. The volume of the Gp5/M binding pocket was calculated with CHECOM algorithm by Yinfo technology. Active pocket of Gp5/M was shown in green. **F** Molecular docking of Gp5/M (PRRSV-2 VR 2332, model 1, alphafold2) with baicalein (Compound CID: 5281605) in a 3D model. **G** Root Mean Square Deviation (RMSD) of baicalein and Gp5/M during MD simulation. **H** Hydrogen bond numbers between baicalein and Gp5/M in 100 ns.

To investigate this uncertainty systematically, a series of experiments were conducted. In these experiments, baicalein was combined with the virus at various concentrations in essential media for one hour at 37 °C. Subsequently, PRRSV was isolated from baicalein using ultrafiltration and then resuspended in essential media to infect MARC-145 cells (Figure 5A). At 48 h post-infection (hpi), the cells were analysed for viral copy number and the relative mRNA expression of ORF7.

The results presented in Figures 5B, C and D demonstrated that co-incubation of baicalein at concentrations of 20, 40, and 80 μg/mL with the virus resulted in a reduced virulence of PRRSV in infecting MARC-145 cells. This indicates a direct interaction between baicalein and PRRSV particles. In contrast, 120 μM of ribavirin did not exhibit direct interaction with PRRSV; rather its effect is attributed to the inhibition of the viral RNA polymerase enzyme during the process of viral replication [36].

To further investigate the mechanism by which baicalein binds directly to PRRSV, we conducted molecular docking and MD simulations of the Gp5/M, envelope protein of PRRSV [37]. The binding pocket volume was predicted to be 9469 Å^3^ based on the structure of GP5/M (PRRSV-2 VR 2332, model 1) [38], using an online tool (Figure 5E). The results from AutoDock indicated that baicalein was stably bound to residues of GP5 (ARG-98, ALA-113, ILE-125), displaying a binding capacity of -6.09 kcal/mol (Figure 5F). The MD simulations revealed that the root mean square deviation (RMSD) of the complex was 0.085 ± 0.029 nm (Figure 5G), and the average number of hydrogen bonds formed throughout the simulation was 2.68 (Figure 5H). These findings suggest that baicalein and Gp5/M can bind to form a stable complex.

### Baicalein improves growth performance and immunity of PRRSV-infected weaned piglets

**Figure 6:**
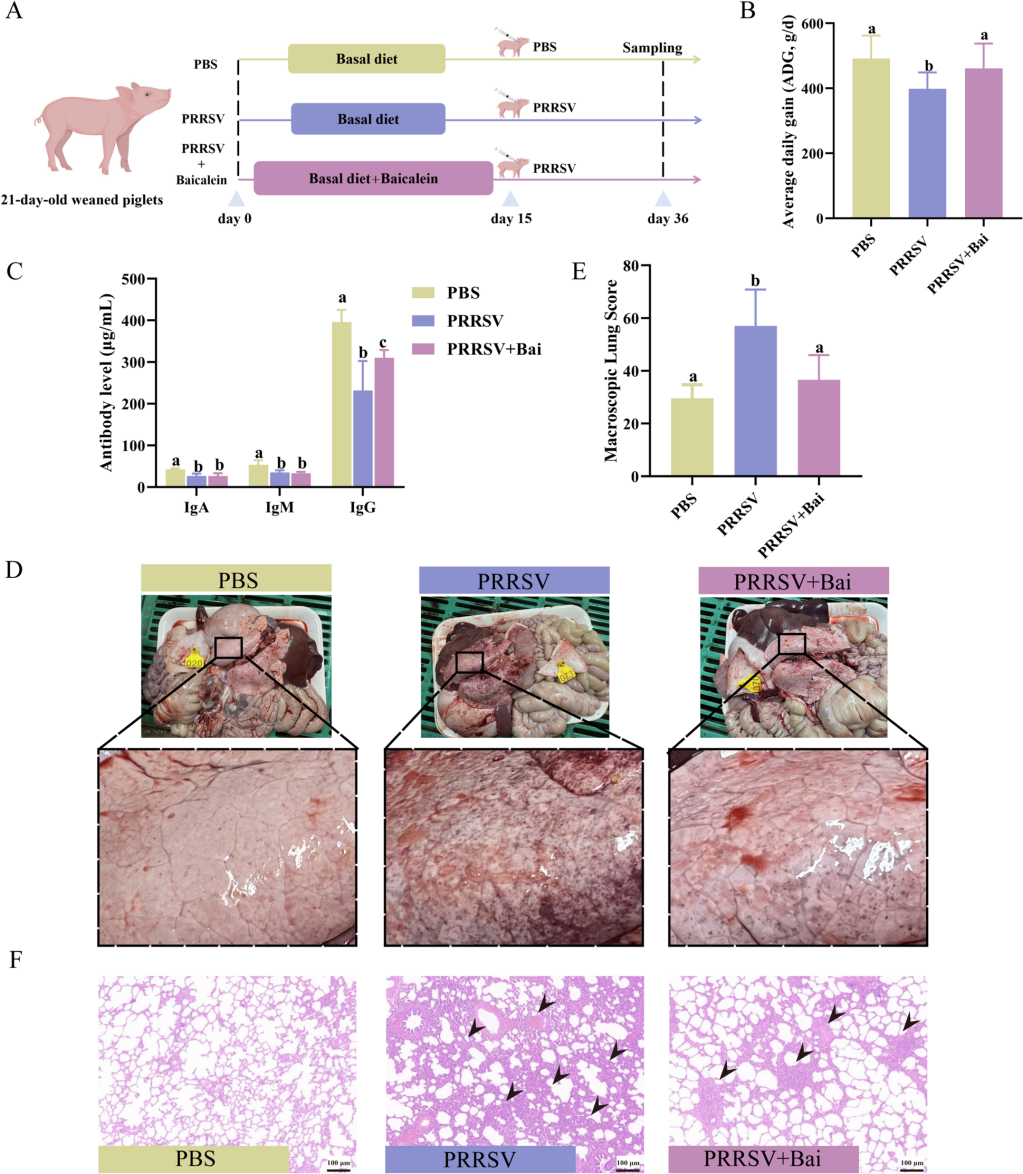
**Baicalein improved growth performance and immunity of PRRSV-infected weaned piglets**. **A** Schematic diagram of experimental design of baicalein inhibiting PRRSV infection in piglets. **B** Average daily gain (ADG) of PRRSV-infected piglets with baicalein treatment. **C** Antibody level of PRRSV-infected piglets with baicalein treatment. **D**-**E** Macroscopic lung lesion changes in PRRSV-infected piglets with baicalein treatment. Macroscopic lungs of piglets in each group were scored by a scoring system. **F** The lungs of piglets were collected, and sections were prepared to stain with haematoxylin and eosin (H&E). Black arrows indicate the histopathological alterations, including progressive loss of lung architecture, thickening of the interlobular septa, and infiltration of lymphocytes (Mean ± SEM, *n* = 3, scale bar = 100 μm).

The experiment was conducted as shown in Figure 6A. Briefly, piglets in the PBS and PRRSV group were fed a basal diet, while those in the PRSV + Bai group received an additional supplement of baicalein for two weeks. On the 15th day of the experiment, each piglet was challenged with either PRRSV or PBS. Three weeks post-infection, serum and lung samples from the piglets were collected for analysis.

The average daily gain (ADG) of piglets in the PRRSV + Bai group (461.11 ± 24.25 g/d) was significantly higher than those in the PRRSV group (398.75 ± 15.74 g/d) (*P* < 0.05, Figure 6B). Additionally, the average daily feed intake (ADFI) also increased significantly from 583.47 g/d (PRRSV group) to 669.57 g/d (PRRSV + Bai group) with baicalein treatment (*P* < 0.05).

Furthermore, ELISA results indicated a significant increase in IgG levels in the PRRSV + Bai group compared to the PRRSV group (*P* < 0.05). This suggests that baicalein may enhance the immunity of PRRSV-infected piglets (Figure 6C).

All piglets were euthanised three weeks after being infected with PRRSV for dissection purposes. Lung samples were collected, photographed, and scored. In the absence of baicalein treatment, the lungs of PRRSV-infected piglets exhibited serious pathological symptoms (Figure 6D, E). The morphological changes in the tissue cells were examined using H&E staining to identify the damage caused by PRRSV infection (Figure 6F). The piglets in the PRRSV group showed significant histopathological alterations in their lungs, including progressive loss of lung architecture, thickening of the interlobular septa, and infiltration of lymphocytes, indicated with black arrows. However, these pathological symptoms were notably alleviated with baicalein treatment (Figure 6F). The findings suggested that baicalein may help reduce the damage from interstitial pneumonia and bronchopneumonia caused by PRRSV infection.

### Baicalein improves the antiviral activity of PRRSV-infected piglets

**Figure 7:**
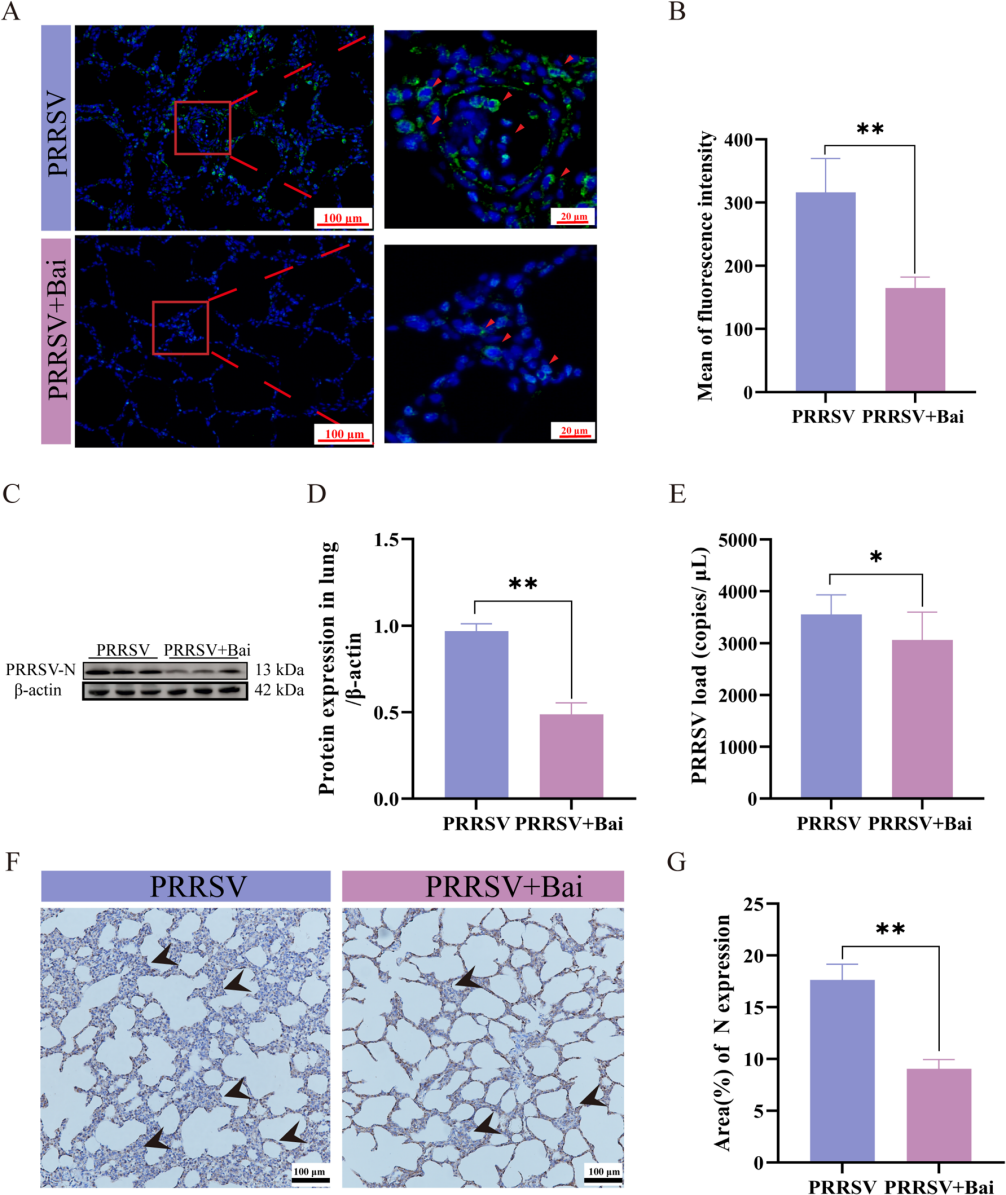
**Baicalein improved antiviral activity of PRRSV-infected piglets**. **A**-**B** IFA analysis of protein N using a rabbit PRRSV N protein polyclonal antibody (1:100, Bioss, Beijing, China). Expression of the protein N is indicated with red arrows. Scale bar = 100 μm; local enlarged images are shown in the right, scale bar = 20 μm (Mean ± SEM, *n* = 3). **C**-**D** Expression of the PRRSV N protein in lung tissues was detected using western blotting (Mean ± SEM,* n* = 3). **E** Viral loads of PRRSV calculated by absolute quantitation. **F**-**G** Expression of PRRSV protein N in lung tissues was detected by IHC with a rabbit PRRSV N protein polyclonal antibody (1:100, Bioss, Beijing, China). Expression of the N protein is indicated with black arrows. Scale bar = 100 μm (Mean ± SEM, *n* = 3). *P < 0.05; **P < 0.01 compared with the PRRSV group.

To investigate the antiviral activity of baicalein, we assessed viral loads and the expression of the N protein. IFA of lung tissues from PRRSV-infected piglets in the PRRSV group revealed a significant presence of N protein fluorescence signals, which appeared predominantly green in color (*P* < 0.05). In contrast, the PRRSV + Bai group exhibited a marked reduction in fluorescence signals for the N protein (Figure 7A, B).

Further analysis through absolute quantitation and western blotting indicated that the baicalein supplement significantly reduced both viral loads and N protein expression in the lungs of PRRSV-infected piglets (*P* < 0.05) (Figures 7C–E). Additionally, immunohistochemistry (IHC) showed that the lungs of PRRSV + Bai group piglets had fewer PRRSV-positive signals (Figures 7F, G). These positive signals were predominantly localised in macrophages, alveolar epithelial cells, and the interstitial regions of the alveoli. Overall, these results demonstrate that baicalein significantly enhances antiviral activity in PRRSV-infected piglets.

### Baicalein improves the anti-inflammatory and antioxidant activity of PRRSV-infected weaned piglets

**Figure 8:**
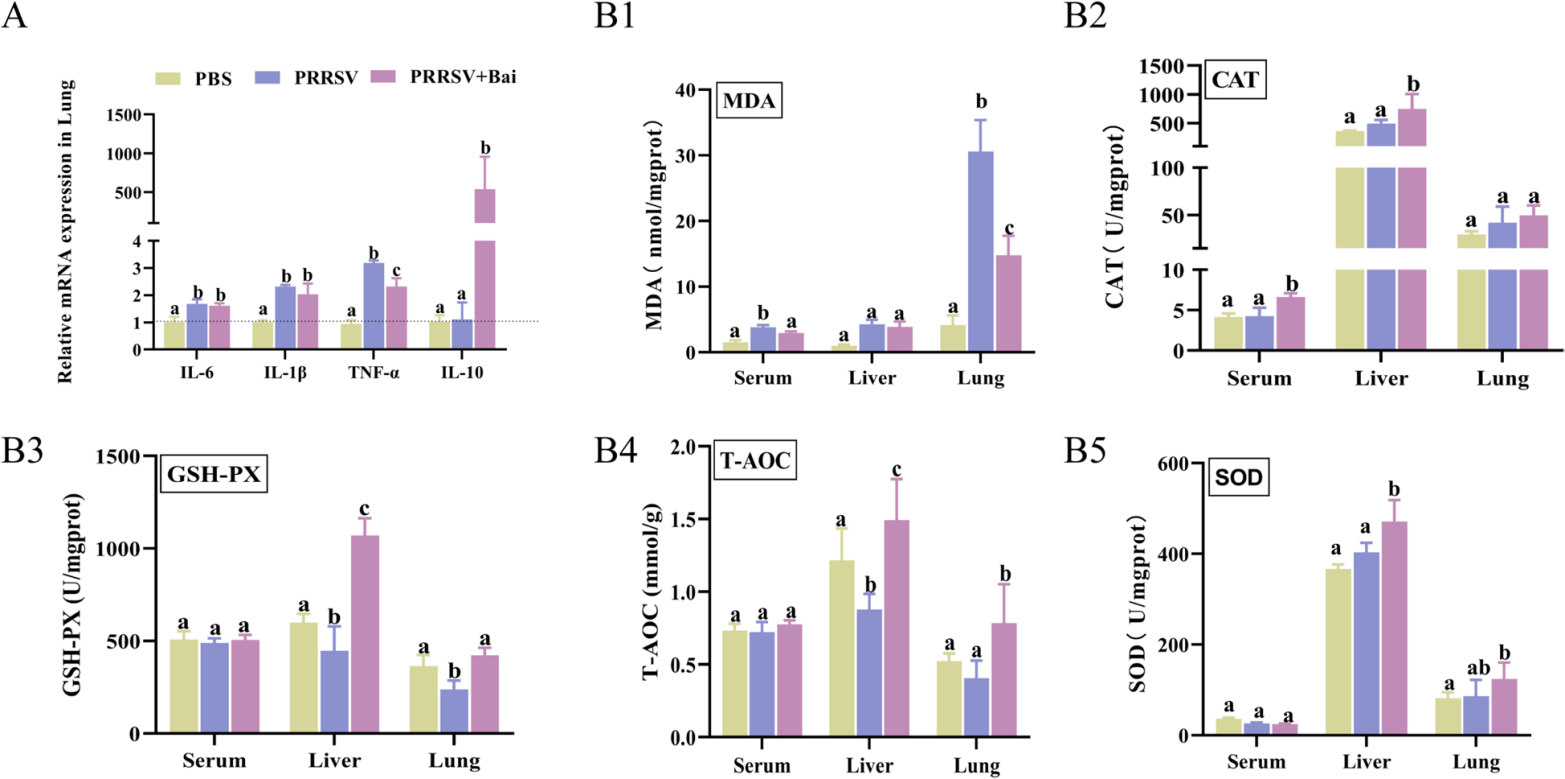
**Baicalein improved anti-inflammatory and antioxidant activity of PRRSV-infected weaned piglets**. **A** The relative expression of inflammatory cytokines in the lung. **B** The antioxidant capacity in serum, liver and lung. The data are expressed as mean ± SD (*n* = 5). *T-AOC* Total antioxidant capacity, *GSH-PX* Glutathione peroxidase, *MDA* Malondialdehyde, *CAT* catalase, *SOD* Superoxide dismutase.

This study investigated the effects of dietary-supplemented baicalein on the secretion of inflammatory cytokines in the lung of PRRSV-infected weaned piglets. The relative mRNA expression of *IL-6*, *IL-1β*, *TNF-α* and *IL-10* was analysed (Figure 8A). While the levels of the pro-inflammatory cytokines *IL-6* and *IL-1β* did not show significant changes with baicalein supplementation, there was a notable decrease in the expression of *TNF-α* and an increase in the anti-inflammatory cytokine *IL-10* (*P* < 0.05).

Additionally, the antioxidant capacity of PRRSV-infected weaned piglets was measured using an antioxidant assay kit. The results (Figure 8B) indicated that in both the liver and lungs of piglets receiving dietary-supplemented baicalein, the level of total antioxidant capacity (T-AOC), SOD and GSH-PX were significantly increased. CAT levels also displayed a significant increase in serum (*P* < 0.05). Furthermore, dietary-supplemented baicalein led to a significant reduction in MDA levels in serum and lungs (*P* < 0.05). These findings suggest that baicalein enhances both the anti-inflammatory and antioxidant capacities of PRRSV-infected weaned piglets.

### Baicalein blocks PRRSV entry via the EGFR-PI3K-AKT pathway

**Figure 9:**
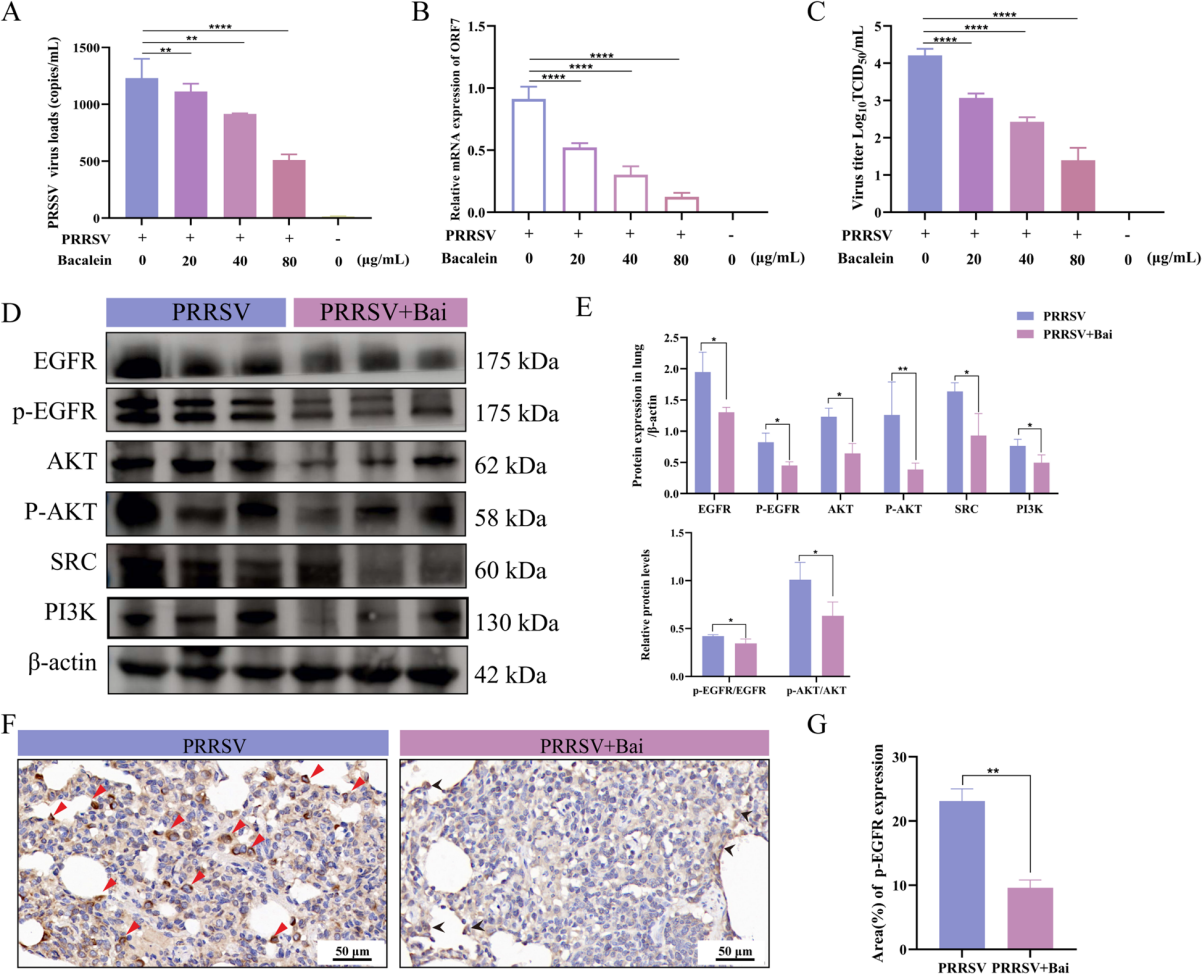
**Baicalein blocked PRRSV entry via the EGFR-PI3K-AKT pathway**. **A** PRRSV copy number calculation using absolute quantification. **B** PRRSV-ORF7 relative expression analysis using qRT-PCR. **C** Virus titres of PRRSV. TCID_50_ of PRRSV were calculated by the Spearman-Kärber method. Each experiment was performed with three biological replicates and three technical replicates. The data are expressed as mean ± SD. **D** Protein expression in the lung of PRRSV-infected piglets by western blotting. **E** Band intensities were subjected to semi-quantitative analysis employing ImageJ software, and statistical significance of the results was analysed utilizing GraphPad Prism software. β-actin served as loading control. **F** Phosphorylation EGFR expression in the lung of PRRSV-infected piglets by IHC. Strongly positive signals are presented with red arrows in PRRSV group and slightly positive signals are presented with black arrows in PRRSV + Bai group. Scale bar = 50 μm. G Area of p-EGFR expression (Mean ± SEM, *n* = 3). *, *P* < 0.05; **, *P* < 0.01, ***, *P* < 0.005, ****, *P* < 0.0001 compared with the only PRRSV treatment group.

The EGFR-PI3K-AKT pathway, which is involved in the rearrangement of the actin cytoskeleton, has been reported to facilitate PRRSV entry [39]. Therefore, we focused on the role of baicalein in modulating the EGFR-PI3K-AKT signalling pathway during PRRSV entry. Our findings indicate that baicalein effectively blocked PRRSV entry into MARC-145 cells in a dose-dependent manner (Figures 9A–C). Additionally, the expression levels of p-EGFR/EGFR, p-AKT/AKT, SRC, and PI3K were significantly down-regulated (*P* < 0.05) (Figures 9D and E). IHC results demonstrated that phosphorylation of EGFR (p-EGFR) in the lungs of PRRSV-infected piglets was primarily localised within macrophages, alveolar epithelial cells, and the interstitial regions of the alveoli (indicated by red arrows in Figure 9F). However, after treatment with baicalein, only slight signals were observed in the interstitial regions of the alveoli (shown as black arrows in Figure 9F). These results suggest that baicalein inhibits PRRSV entry by suppressing the EGFR-PI3K-AKT signalling pathway.

## Discussion

Porcine reproductive and respiratory syndrome (PRRS) presents a significant challenge to the global swine industry. As a major contributor to porcine respiratory disease complex (PRDC), PRRS often weakens the porcine immune system and leads to severe respiratory symptoms in weaned piglets [40]. The PRRS virus (PRRSV) is a highly mutated RNA virus with considerable genetic variability, making the development of a reliable and effective vaccine difficult. [41]. Additionally, the use of antiviral drugs in livestock raised for food is prohibited in China due to concerns about drug residues [5]. As a result, developing a potent antiviral treatment is essential for managing PRRSV infections.

Baicalein, a flavonoid compound derived from *Scutellaria baicalensis*, has garnered attention for its potential to inhibit virus replication [10, 11, 14]. In this study, we investigate the potential targets of baicalein against PRRSV using network pharmacology and molecular docking techniques. Network pharmacology, which relies on extensive biomedical data, enhances research into the functional targets of plant extracts [42]. The results from our network pharmacology analysis indicated that baicalein's potential targets in combatting PRRSV include EGFR, TP53, STAT3, SRC, MMP9, INS, and ESR1 (degree > 31). These targets were found to be enriched in the MAPK and Ras signalling pathways. Our findings suggest that baicalein inhibits PRRSV infection by regulating cellular oxidative stress, inflammation, and other pathological responses. This network pharmacology analysis has provided a framework for our research, helping shed light on the antiviral mechanisms of plant extracts.

Molecular docking studies have shown that EGFR is a key target of baicalein in the fight against PRRSV. This supports the hypothesis that certain core proteins are crucial for baicalein’s anti-PRRSV activity. EGFR, a receptor tyrosine kinase (RTK), activates tyrosine kinases when bound to ligands, triggering various intracellular signalling pathways, such as MAPK-ERK, PI3K-AKT, Ras/Raf/ERK1/2, and JAK-STAT [43]. Our network pharmacology results also identified these signalling pathways. It is well-established that many viruses, including herpes simplex virus 1 [44], spleen and kidney necrosis virus [45], influenza a virus [46], utilise EGFR-mediated signalling pathways for entry into host cells. Notably, the EGFR-PI3K-AKT signalling pathway is involved in regulating cofilin activation, which is critical for actin cytoskeletal rearrangement and, consequently, for PRRSV entry [39]. Our findings reveal that baicalein treatment resulted in lower expression levels of p-EGFR and its downstream proteins. This consistent observation suggests that baicalein inhibits PRRSV entry by disrupting the EGFR-PI3K-AKT signalling pathway.

The PI3K-AKT pathway plays a crucial role in various cellular activities, including cell growth, migration, survival, and vesicle transport. It is also a key mediator in the regulation of actin cytoskeleton rearrangement and cell migration [47]. When a virus binds to its cellular receptor, EGFR is activated, initiating a signalling cascade that promotes the phosphorylation of cofilin through the PI3K-Akt pathway. This process ultimately leads to the rearrangement of actin and the endocytosis of the virus into the cell [44]. Although the specific mechanisms by which baicalein blocks PRRSV entry are not fully understood, one possible explanation involves its ability to inhibit EGFR phosphorylation. This occurs through baicalein’s binding to EGFR, which reduces actin rearrangement, as suggested by MD simulations and western blotting results.

In vitro experiments have shown that baicalein, similar to Sanggenon C and artesunate, exhibits anti-PRRSV activity. Baicalein demonstrated inhibitory effects during the pre-treatment, co-treatment, and post-treatment stages. This suggests that baicalein could play a dual role in both the prevention and treatment of PRRSV infection. However, unlike the other two plant extracts, which do not bind directly to PRRSV, baicalein significantly inhibits PRRSV in vitro [48, 49].

This observation implied that baicalein may possess effective antiviral properties not only through interactions with cells but also by directly binding to specific structural proteins of PRRSV. These proteins include GP2a, GP2b, GP3, GP4, GP5, the matrix protein M, and the nucleocapsid protein N [50–52]. This potential was further supported by the results of molecular docking and MD simulations of baicalein with Gp5/M conducted in this study.

The anti-PRRSV effect of matrine has been documented, as it directly inactivates the virus in vitro and influences the expression of the PRRSV N protein [53]. Additionally, a previous study found that 1.05% peppermint extract can directly kill the virus and can be used as a disinfectant to eliminate African Swine Fever Virus [54]. Baicalein shows promise for disinfection processes against PRRSV, offering a solution that is both safe and effective for humans and animals.

The anti-PRRSV effect of baicalein has been established through both animal experiments and studies with MARC-145 cells, which are permissive to PRRSV [42]. The results of this study showed that dietary supplementation with baicalein reduced the copies of the PRRSV-N gene, improved the growth performance and acquired immunity of weaned piglets, and alleviated the damage caused by PRRSV infection, including interstitial pneumonia and bronchopneumonia. Similarly, other plant extracts, such as *Cryptoporus volvatus* and Sanggenon C, also demonstrated a resistance to PRRSV infection in weaned piglets, comparable to baicalein [55, 56]. Furthermore, treatment with baicalein significantly decreased both PRRSV viral loads and the expression of the N protein, indicating that baicalein could be a potential candidate drug for the treatment of PRRSV.

The antiviral mechanisms of plant extracts generally include directly killing the virus [57], preventing the virus from entering host cells [58], inhibiting viral multiplication [59], and boosting the host’s immune response [60]. When viruses invade host cells, they trigger a strong immune reaction, which can create challenges for the organism’s innate defence system in effectively combating multiple viruses. It is important to explore how plant extracts exert their antiviral effects by influencing the host immune system [60].

In our study, we found that the IgG levels in PRRSV-infected piglets significantly increased when supplemented with baicalein, although these levels remained lower than those in the PBS group. This suggests that baicalein somewhat enhances the acquired immune response in PRRSV-infected piglets. The innate immune system plays a crucial role in eliminating viruses and restoring homeostasis after inflammation triggered by virus infections [61].

In line with previous findings on the anti-inflammatory properties of baicalein [62], this study observed a reduction in pro-inflammatory cytokines (*TNF-α*) and an increase in anti-inflammatory cytokines (*IL-10*) when baicalein was administered. Additionally, cellular oxidative stress is significantly heightened in cells infected with PRRSV [63]. Baicalein plays a similar role in alleviating oxidative stress as xanthohumol [63] and artesunate [48].

The observed increase of T-AOC, GSH-Px, CAT, along with a decrease in MDA, suggests that baicalein inhibits PRRSV infection through an antioxidant pathway. Additionally, the lower expression of the N protein indicates that baicalein may activate the interferon signalling pathway to inhibit PRRSV replication, which aligns with the mechanism by which Toosendanin acts against PRRSV [64].

**Figure 10:**
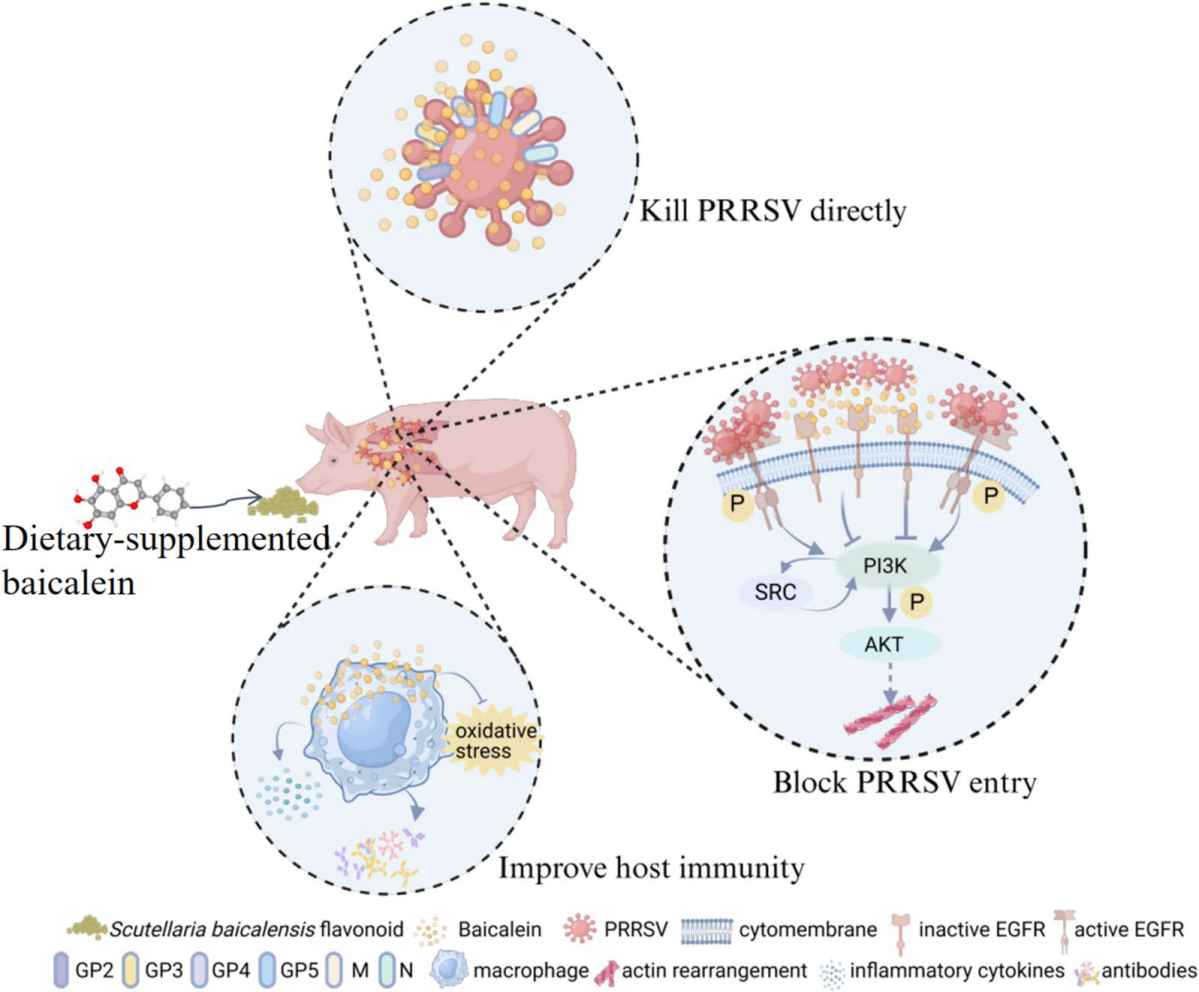
**Scheme summarizing the inhibitory effect of baicalein on PRRSV infection**. The underlying antiviral mechanisms of baicalein including binding to PRRSV structural protein to directly kill the virus, inhibiting EGFR-PI3K-AKT pathway to block PRRSV entry, and boosting host immunity to inhibit PRRSV infection. The figure was generated by BioRender with authorised consent for publication

In conclusion, this study utilised a combination of network pharmacology, molecular docking, MD simulation, cell experimental validation, and animal experiments for the first time to investigate the effectiveness and mechanism of baicalein against PRRSV infection. The findings identified potential targets of baicalein in combatting PRRSV and clarified its antiviral mechanisms. Specifically, baicalein was shown to bind to structural proteins of PRRSV, directly reducing viral infectivity. Additionally, it inhibits the EGFR-PI3K-AKT pathway, which helps block the entry of the virus, and enhances the host’s immune response against PRRSV infection (Figure 10). This study supports the potential of baicalein as a promising pharmaceutical candidate for the prevention and control of PRRS and provides new insights into the antiviral mechanism of traditional Chinese medicine.

## Supplementary Information

**Additional file 1: HPLC chromatogram analysis of baicalein. The retention time of baicalein was 17.522 min with the purity of 96.7%**.

**Additional file 2: Dietary ingredient composition in animal experiment**.

**Additional file 3: Primers used in this study**.

## Data Availability

All data generated during this study are included in this published article and its supplementary information files.
