# Supplementary material for: Baicalein inhibits PRRSV through direct binding, targeting EGFR, and enhancing immune response
Source: Vet Res. 2025 Jan 20;56:16. doi: 10.1186/s13567-024-01440-5 (PMC11748510; doi:10.1186/s13567-024-01440-5)
Supplement: Supplementary file 3 — Additional file 3: Primers used in this study. [file 13567_2024_1440_MOESM3_ESM.docx]

**Additional file 3 Primers used in this study**

| Genes |  | Sequences (5’-3’) | GenBank Accession |
| --- | --- | --- | --- |
| *IL-6* | Forward | TACATCCTCGGCAAAATC | NM_214399 |
|  | Reverse | TCTCATCAAGCAGGTCTCC |  |
| *IL-1β* | Forward | CTCCAGCCAGTCTTCATTGTTC | NM_214055.1 |
|  | Reverse | TGCCTGATGCTCTTGTTCCA |  |
| *IL-10* | Forward | CTGCATCCACTTCCCAACCA | NM_214041.1 |
|  | Reverse | CGGCATTACGTCTTCCAGGT |  |
| *TNF-α* | Forward | CCAATGGCAGAGTGGGTATG | [NM_214022.1](https://www.ncbi.nlm.nih.gov/entrez/viewer.fcgi?db=nucleotide&id=47522865) |
|  | Reverse | TGAAGAGGACCTGGGAGTAG |  |
| *ORF 7* | Forward | CCAGCCGGTCAATCAGCT | MK359282.1 |
|  | Reverse | GGCTTCTCCGGGCTTTTCT |  |
